# Automated Detection of Periodontal Bone Loss in Two-Dimensional (2D) Radiographs Using Artificial Intelligence: A Systematic Review

**DOI:** 10.3390/dj13090413

**Published:** 2025-09-08

**Authors:** Alin M. Iacob, Marta Castrillón Fernández, Laura Fernández Robledo, Enrique Barbeito Castro, Matías Ferrán Escobedo Martínez

**Affiliations:** 1 Department of Surgery and Medical-Surgical Specialities, School of Medicine and Health Sciences, University Dentistry Clinic, University of Oviedo, 33006 Oviedo, Spain; uo272130@uniovi.es (A.M.I.); uo283277@uniovi.es (M.C.F.); uo282680@uniovi.es (L.F.R.); 2 Central University Hospital of Asturias, 33003 Oviedo, Spain; uo290649@uniovi.es

**Keywords:** artificial intelligence, periodontal bone loss, convolutional neural network, periapical radiograph

## Abstract

Artificial intelligence is an emerging tool that is being used in multiple fields, including dentistry. An example of this is the diagnosis of periodontal bone loss by analyzing two-dimensional (2D) radiographs (periapical, bitewing, and panoramic). **Objectives**: The objectives of this systematic review are to bring together the existing evidence and evaluate the effectiveness of the different artificial intelligence architectures that have been used in recent studies. **Materials and Methods**: This work has been carried out following the PRISMA criteria and has been recorded in PROSPERO (ID = CRD 42025640049). We searched six different databases, and the results were filtered according to previously established inclusion and exclusion criteria. We extracted data independently by three review authors and analyzed the risk of bias of the studies using the QUADAS-2 test, calculating Cohen’s kappa index (κ) to measure the agreement between assessors. **Results:** We included 20 diagnostic accuracy studies according to the inclusion and exclusion criteria, published between 2019 and 2024. All included studies described the detection of periodontal bone loss on radiographs. **Limitations:** One of the main limitations identified was heterogeneity in the indices used to assess the accuracy of models, which made it difficult to compare results between studies. In addition, many works use different imaging protocols and X-ray equipment, introducing variability into the data and limiting reproducibility. **Conclusions:** Artificial intelligence is a promising technique for the automated detection of periodontal bone loss, allowing the accurate measurement of bone loss, identifying lesions such as apical periodontitis and stage periodontitis, in addition to reducing diagnostic errors associated with fatigue or inexperience. However, improvements are still required to optimize its accuracy and clinical applicability.

## 1. Introduction

Periodontal bone loss is a common manifestation of both periodontal diseases and dental lesions, and it can occur in different locations depending on the origin of the pathological process. This bone destruction can be observed at the apical level, typically associated with pulp infections and apical periodontitis [1,2], as well as in the marginal regions of the alveolar bone, which is usually linked to chronic periodontal inflammation [3,4].

Periodontal disease (PD) is characterized by pathological changes that occur in the periodontium, including the gingival tissue, alveolar bone, cementum, and periodontal ligament [5].

Periodontal disease (PD) is a multifactorial inflammatory condition associated with the accumulation of dental plaque, leading to the progressive destruction of the periodontal ligament and bone [2]. It involves complex interactions between bacterial pathogens and factors such as smoking, triggering an immune response that damages the periodontium [6,7]. Its clinical features include gingival inflammation [8], bleeding on probing [7], periodontal pockets [8], clinical attachment loss [9,10], dental mobility, pathological migration [11], alveolar bone loss [12], and ultimately, tooth loss [7].

Early diagnosis of periodontal disease is crucial, as its progression can lead to tooth loss and functional alterations. Periodontal examination is key, as the periodontal probing chart reflects pocket depths, clinical attachment loss, and gingival recessions [11]. Additionally, periapical radiographs, bitewing radiographs, and panoramic radiographs are used to assess bone loss [9].

In recent years, artificial intelligence (AI) has become widely used across various fields, including learning and logical reasoning, with the goal of mimicking functions of the human mind. AI has given rise to two more specific branches: “Machine Learning” and “Deep Learning.” One notable example is Convolutional Neural Networks (CNNs), which are models that recognize complex patterns similar to the human brain [13].

CNNs are a type of deep neural network specifically designed to process grid-like data, such as images. Unlike traditional methods, CNNs can autonomously extract features without the need for human intervention [14].

In dentistry, AI is used to diagnose diseases, plan treatments, and detect pathologies such as caries, periodontal lesions, root fractures, and maxillary sinusitis [12], with the primary goal of automatically identifying pathologies, diseases, or anatomical structures and evaluating their severity [5]. Intraoral radiographs and panoramic radiographs are commonly used to diagnose periodontal disease, with the latter being useful for its wider field of view and ease of use [15]. Cone Beam Computed Tomography (CBCT) is also employed, though less frequently due to its higher radiation dose and limited availability [16,17]. The use of AI in radiographs saves time, reduces discrepancies between examiners, and provides reliable diagnoses, even for non-specialist physicians [18,19,20,21,22,23].

This review aimed to synthesize current evidence on AI-based methods for detecting periodontal bone loss in radiographs, evaluate their diagnostic accuracy, and explore their clinical applications in periodontics.

## 2. Materials and Methods

This systematic review was conducted following the PRISMA (Preferred Reporting Items for Systematic Reviews and Meta-analyses) guidelines proposed by Page et al. in 2020 [24]. This study was registered in PROSPERO (International Prospective Register of Systematic Reviews) with ID = CRD42025640049. This manuscript used NLP (ChatGPT-3.5) for final revision and proofreading an entirely human-generated text, but no other use of AI was made.

To structure the search and selection of studies included in this review, the following “PICOS” criteria were defined:

Population (P): Human patients receiving dental care with radiographic images for periodontal health assessment.

Intervention (I): Application of AI algorithms for the automatic detection of periodontal bone loss in 2D radiographs.

Comparison (C): Detection performed by human experts (e.g., dentists or radiologists) using manual radiographic interpretation methods.

Outcome (O): Quantification of the method’s accuracy using indices such as precision, sensitivity, and specificity of AI in detecting periodontal bone loss compared to human experts. Additional outcomes may include time efficiency and inter-observer consistency.

Study Design (S): Diagnostic accuracy studies, observational studies, or randomized clinical trials.

### 2.1. Inclusion and Exclusion Criteria

Thanks to recent advances in these techniques improving diagnostic accuracy, studies published since 2018 were included, evaluating periodontal bone loss in patients receiving dental care through radiographs, where AI models were applied for automatic detection, comparing the results with interpretation or supervision by human experts. Diagnostic accuracy studies, observational studies, and randomized clinical trials were considered, provided they reported quantitative indices such as sensitivity, specificity, and precision of AI, as well as its efficiency and inter-observer consistency. The inclusion of articles was limited to studies published in English or Spanish.

Studies were excluded if they did not use radiographic images to assess periodontal health, did not apply AI for detecting bone loss, did not include comparisons with human experts using traditional methods, did not report results quantifying performance, efficiency, or consistency, or had designs other than diagnostic, observational studies, or controlled trials. Also excluded were studies in non-reviewable languages, those with low methodological quality, literature reviews, letters to the editor, case series, and studies not conducted on humans.

### 2.2. Search Strategy

A bibliographic search was conducted between 27 October and 10 November 2024, and updated on 10 March 2025. The search was performed using the following databases: PubMed, Web of Science, Scopus, Embase, and Cochrane. A gray literature search was also carried out in OpenGrey, including reports, theses, technical documents, and other unconventional sources of scientific information.

The search strategy was developed using various combinations of keywords with the Boolean operators “OR” and “AND.” The different syntaxes for each database are presented in Table 1.

### 2.3. Data Extraction

The data were extracted independently by three reviewers (M.C.F., L.F.R., and A.M.I.) and compiled into a standardized table.

For each article, we included the author and year of publication, full original title, study type and objective, type of radiograph used, sample size (number of radiographs divided into training, validation, and test sets), number of patients, AI technique, reference standard for comparison, indices used for model quantification, and index scores. We were limited to reporting and analyzing the available data, as we did not have access to additional information to complete the missing datasets.

### 2.4. Risk of Bias Assessment

Based on the recommendation of the JBI [25] and the work of Ma et al. [26], the QUADAS-2 (Quality Assessment of Diagnostic Accuracy Studies 2) tool was independently applied by the authors (A.M.I. and M.F.E.M.) to analyze the included studies, with the aim of considering individual sources of bias risk. The QUADAS-2 tool included the following domains: Risk of Bias (evaluating patient selection, indices calculated for system accuracy, reference standard for comparison, and study flow and timing) and applicability criteria (evaluating patient selection and reference standard used for segmentation) to deduce an overall risk of bias for the study. Prior to the assessment process, the reviewers agreed on specific criteria that should be applied for the inclusion or exclusion of any study from the review; this criterion was then consistently applied across all studies.

The risk of bias and applicability of the included studies were independently assessed by two investigators using the QUADAS-2 tool, which classifies studies into three levels (low, moderate, and high). However, in this work, only the categories “low” and “unclear” were used, with the latter grouping studies with potential risk of bias and those with insufficient information for a clear judgment.

To measure inter-rater agreement, Cohen’s kappa (κ) index was used, categorizing the evaluations as “low”, “high”, and “unclear,” calculating it for each QUADAS-2 domain. The interpretation of the results followed the criteria of Landis and Koch [27], which classify κ values from slight agreement (<0.20) to almost perfect agreement (>0.80).

## 3. Results

### 3.1. Study Selection

**Figure 1:**
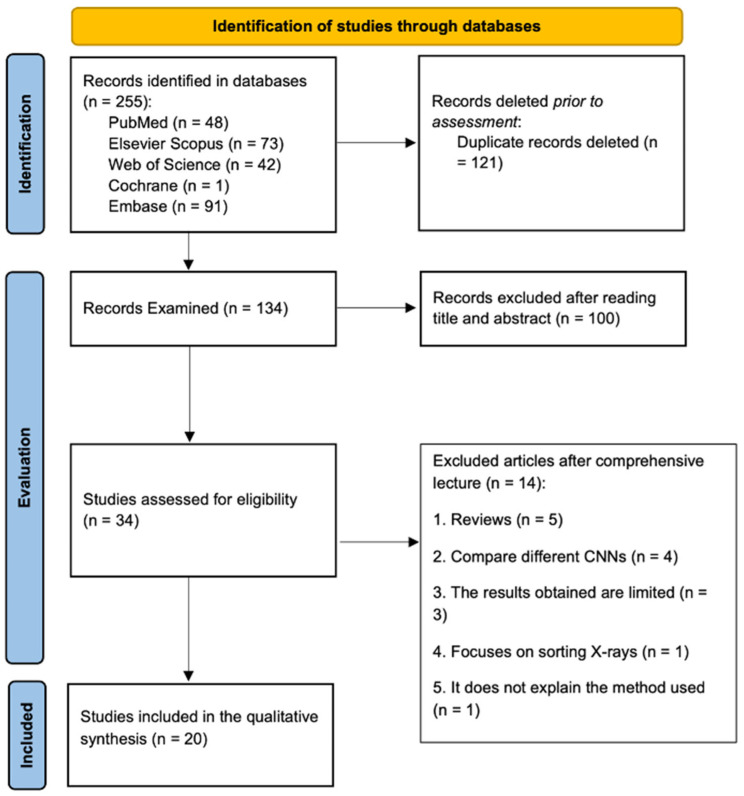
PRISMA Flowchart showing the process of study selection.

Our search yielded 255 results, none of which were gray literature. After checking the titles and authors of the articles obtained using the formula in the different databases, 121 duplicates were removed. Next, the titles and abstracts of the remaining 134 articles were read, and 34 potentially relevant articles were selected for full-text reading. After the full-text reading of these 34 articles and the application of the inclusion and exclusion criteria, 20 articles were selected. The process is illustrated in the PRISMA flow diagram (Figure 1).

### 3.2. Study Characteristics

The 20 included studies (Table 2) were diagnostic accuracy studies written in English from 2019 to 2024 [5,18,23,28,29,30,31,32,33,34,35,36,37,38,39,40,41,42,43,44]. These studies included imaging tests, using panoramic radiographs, periapical radiographs, and bitewing radiographs. All studies employed CNNs or a variant of them.

### 3.3. Study Focus

A total of 11 studies focused on “periodontal bone loss” [5,18,31,32,34,37,38,40,41,43,44]. Additionally, one study examined the “periodontal disease classification” separately [28], and another study investigated both “periodontal disease classification” and “periodontal bone loss” [35]. On the other hand, four studies focused on “bone loss in apical periodontitis” [23,29,30,33]. Finally, three other studies had a broader focus, covering various pathologies such as caries and periodontitis [16], missing teeth, caries, fillings, prosthetic restorations, endodontics, residual roots, periapical lesions, and periodontal bone loss [39], and even alterations in periodontal bone height and impacted third molars [42]

### 3.4. Radiographs Employed

The radiographs studied were, in two of the studies, periapical and bitewing radiographs [32,38], while eleven studies evaluated panoramic radiographs [5,18,23,28,31,33,34,39,41,42,43]. Seven studies evaluated only periapical radiographs [29,30,35,36,37,40,44]. The number of radiographs varied from 30 [39], to 103,914 [30]. In total, 141,166 radiographs were used, divided into 20,942 panoramic radiographs and 120,224 periapical and bitewing radiographs.

The radiographs were divided into three groups: training, validation, and test; twelve studies clearly specified the amounts for each group [5,18,23,29,34,35,37,38,41,42,43,44], while seven studies did not specify this division [28,30,32,33,36,39,40]. In the study by Kong et al. [31], a random sample division was used.

### 3.5. Patient Sample

The number of patients was a parameter not clearly expressed in all the studies. Twelve of the included studies did not specify the sample size of patients [5,23,28,29,30,31,33,34,35,41,42,43], while eight studies did report it [18,32,36,37,38,39,40,44]. The studies employed a range from 30 patients [39] to 10,489 patients [38]. The wide variation in dataset sizes, from as few as 30 images to more than 100,000, may partly explain differences in reported performance. Small datasets risk overfitting and reduced generalizability, whereas larger datasets tend to provide more stable results.

### 3.6. AI Technique Employed

The techniques for detecting periodontal bone loss were highly heterogeneous across studies. Each study used different CNN architectures, including ResNet, used by Li et al. [36]; a two-stage periodontal disease detection network based on CNN was employed by Kong et al. [31]; Kim et al. [28] studied a UNet-CVAE network, and Kearney et al. [38] chose a “generative adversarial restoration network with partial con-volutions.” Danks RP et al. [40] opted for a “deep neural network with a sandglass architecture.”

Next, Chen et al. [32] used a CNN with “VGG-16” and “U-Net” architecture, while Ayyildiz et al. [34], chose a transfer learning (TL) method based on CNN, and Kim et al. [43], analyzed a network called “DeNTNet.” Chen et al. [35] used “Mask-RRNC” and “U-Net.” Saylan et al. [5] selected a “Yolo-v5” model, and Bayrakdar et al. [41] decided on the CNN “Google Net Inception v3.”

On the other hand, Zadrozny et al. [39] and Nagareddy et al. [33] used Diagnocat as their CNN; Icoz et al. (28) employed computer-aided diagnosis (CAD) based on Yolo (CNN), and Liu et al. [29] and Boztuna et al. [23] chose YoCNET (Yolov5 + ConvNeXt) and U2-Net as their CNNs, respectively.

Finally, four authors [18,37,42,44] did not specify the CNNs they used in their studies.

### 3.7. Reference Standard

In all cases, manual detection was used as the reference standard to assess the accuracy of the AI models employed in detecting this condition, except for the study by Kim et al. [28], which compared the diagnostic accuracy of the studied model with other deep learning systems supervised by a human operator. The radiographs were analyzed by multiple professionals, including general dentists, periodontists, maxillofacial surgeons, and radiologists, to later compare their observations with the analyses performed by the different AI models.

### 3.8. Index Used

To evaluate the effectiveness of the models, different indices were used, with the most common being the F1 score, accuracy, precision, sensitivity, and specificity. Other indices such as the Dice-Sørensen Coefficient (DICE) and Area Under the ROC Curve (AUROC) were also used. The F1 score was the most widely used parameter to quantify model performance, as it combines precision and sensitivity into a single measure [5,18,23,28,29,31,32,33,34,35,36,37,41]. The second most used parameter was precision, which measures the proportion of true positives among all positive predictions made [5,18,23,28,29,31,32,33,34,35,36,37,41]. Another parameter calculated was sensitivity, also known as “recall,” which indicates the model’s ability to correctly identify all instances [5,28,29,30,34,35,36,37,39,41,42,43,44]. In studies like that of Boztuna et al. [23], DICE was used to quantify performance. Studies such as those by Ayyildiz et al. [34], Chen et al. [35], and Kim et al. [43] used AUROC to evaluate the discriminative ability of their classification models. Other indices like positive predictive value (PPV) and negative predictive value (NPV) were also calculated [36,43].

### 3.9. Accuracy of the Studies

The accuracy reported in the included studies varied considerably, reflecting differences in model architecture, the quality of training data, and the specific diagnostic tasks addressed. For example, Kim et al. [28] and Boztuna et al. [23] reported moderate accuracy scores of 0.827 and 0.788, respectively, which are consistent with typical ranges for diagnostic models dealing with complex dental structures.

In contrast, Liu et al. [29] and Xue et al. [18] reported significantly higher accuracy scores of 0.9093 and 0.9178, respectively, indicating superior performance of their models. This higher accuracy could be related to the use of more advanced convolutional architectures, such as ConvNeXt, or to training datasets that were more carefully developed to reflect greater clinical variability. In particular, the approach of Xue T et al. [18] focusing on early-stage diseases highlights the importance of early detection in the management of periodontitis.

Additionally, Nagareddy et al. [30] presented a more complex analysis, which included accuracy estimates for multiple evaluators, including two radiologists (R1 and R2) and the AI system.

Overall, larger datasets and intraoral radiographs (periapical and bitewing) were associated with higher diagnostic accuracy, while panoramic radiographs generally showed lower performance due to image distortion and overlapping structures. Outlier results often reflected differences in dataset size, annotation quality, or CNN architecture.

### 3.10. Risk of Bias Assessment Results

The risk of bias for each article was independently assessed by the researchers using the QUADAS-2 tool, and the results for each domain were recorded in tables. The evaluated domains included patient selection, the indices calculated for system accuracy, the reference standard for comparison, and study flow and timing. The results from the QUADAS-2 tool reflected a low overall risk of bias, with methodological uncertainties regarding applicability criteria in three articles and a risk of bias due to patient selection in seven articles.

The inter-rater agreement analysis, presented in Table 3, showed a good level of agreement in most of the domains assessed with the QUADAS-2 tool. In the bias evaluation, substantial agreement was observed in the domains of “patient selection” (κ = 0.8864) and “calculated indices” (κ = 0.8276), while the domains of “reference standard” and “study flow” showed moderate agreement (κ = 0.7727). Regarding applicability, moderate agreement was found both in “patient selection” (κ = 0.7727) and in the “reference standard” (κ = 0.6429), reflecting reasonable consistency between evaluators.

## 4. Discussion

Given the growing use of AI applications in dentistry, the aim of this systematic review was to analyze the current evidence and effectiveness of automated techniques for detecting periodontal bone loss in dental radiographs using AI, focusing on their diagnostic accuracy, efficacy, and clinical applications.

The assessment of alveolar bone loss is essential for diagnosing periodontal disease and establishing its prognosis. The visual analysis performed by a dentist or radiologist can be enhanced with the use of AI, resulting in lower error rates compared to human observers, as well as faster results [5,45].

According to the articles included in this systematic review, it has been observed that the application of AI for detecting periodontal bone loss has shown high performance, despite the different algorithms used in the various studies, as well as the type of radiographic technique.

### 4.1. Radiographic Techniques

Regarding radiographic techniques, we found that different studies used panoramic radiographs [5,18,23,28,31,34,39,41,42,43], periapical radiographs, or a combination of periapical and bitewing radiographs [32,38].

Different types of radiographs, such as panoramic radiographs, periapical radiographs, and bitewing radiographs, have inherent characteristics that affect the input data for AI models [34,39,42]. Panoramic radiographs, for example, provide a wider field of view than periapical radiographs but have lower resolution for individual teeth, making them more prone to distortion, overlapping structures, and blurred dental edges [5,31,35,37]. This can make it more difficult to detect local changes such as periodontal bone loss [35]. Authors like Kong et al. [31] note that diagnostic accuracy may be lower in panoramic radiographs compared to intraoral radiographs [31]. Nevertheless, images considered of low quality due to acquisition errors or artifacts are typically excluded from the study datasets [5,23,30,34].

Of the selected articles, six did not specify the sample division used for training, testing, and validating the architecture, resulting in a loss of information regarding the model’s performance [28,30,32,33,39,40]. It was also found that the sample sizes varied widely, ranging from 30 radiographs [30,39] to 103,914 radiographs [38]. A small sample size in model training can lead to overfitting errors, reduced accuracy, and poor performance when applying the model to new datasets with different characteristics [18,35,39]. By contrast, larger datasets generally enhance robustness and stability of the models, although they may still be limited if derived from a single source.

### 4.2. AI Architectures/Models Used

Most of the studies applied CNN learning mechanisms or their modifications for AI. It has been found that the most commonly used AI techniques were U-Net and YOLO, both as standalone architectures [5,23,33,43], and in combination with others [28,29,32,35]. Additionally, it is worth noting that Diagnocat^®^ was the third most used model [30,39].

Although most of the articles provide detailed explanations of the functioning of the models, others directly addressed the results, such as the study by Nagareddy et al. [30].

The most used evaluation indices among the 20 studies are the F1 score, specificity, sensitivity, precision, accuracy, and AUROC [5,7,8,9,11,12,13,14,15,16,17,18,19,20,21,22,23]. It is also worth noting the use of the intraclass correlation coefficient (ICC) among the included studies for internal validity [12,22].

### 4.3. Comparison with Traditional Methods

The effectiveness of automated periodontal bone loss detection models is evaluated by comparing their results with those obtained through manual methods, considered the reference standard [18,23,31,33,35,36,43,44]. In most studies, the images are initially inspected by a group of examiners, typically dentists [18,23,29,35,36,37,43,44]. In the study by Li et al. [36], dentists initially assessed the bone loss of each tooth, and two weeks later, 400 teeth from the test set were re-evaluated to compare the results with the deep learning model. A final review was conducted two months later to confirm the findings [36]. Since the clinical diagnosis of bone loss can be subjective and vary between researchers, radiographs with discrepancies in interpretation were discarded, as noted in the study by Alotaibi et al. [37]. In some cases, such as in the study by Boztuna et al. [23], an additional examiner was needed to resolve discrepancies between the dentists. In other studies, once annotations were made by the study dentists, they were supervised by other examiners to confirm the results, as seen in the work of Xue et al. [18]. Annotations can also be made with the support of labeling modules; for example, in the study by Saylan et al. [5], CranioCatchâ was used to determine the level of bone loss in each radiograph before inputting them into the architecture to be trained [5].

AI models first perform preprocessing to isolate the teeth and locate the areas of interest for diagnosis. In the model proposed by Liu et al. [29], the teeth were automatically identified, the surrounding area was expanded, and each area was classified using ConvNeXtâ based on the presence or absence of lesions [29]. These models include a training, validation, and testing phase to obtain the final diagnosis. An example is the AI method described by Kim et al. [43], in which the region of interest was first segmented and then periodontal bone loss lesions were predicted for each tooth [41]. To optimize the model’s performance, independent classifiers were developed for premolars and molars, with the results integrated in a final stage to generate the overall prediction [43].

### 4.4. Advantages of AI-Based Methods

Studies in this field are essential, as AI systems offer significant advantages over traditional methods. Dental radiographs allow for rapid, precise, and automated evaluations of periodontal status [46]. This potentially overcomes the limitations of conventional methods, where the limited time available for interpreting radiographs can increase costs and compromise the quality of patient care [5]. Clinical decisions made by dentists are inherently subjective, exhibit greater interobserver variability, and require more time, which increases the risk of diagnostic errors [5]. AI systems enable the standardization of these decisions, reducing interobserver variability [41,46]. Furthermore, deep learning models have shown greater sensitivity and specificity in detecting lesions compared to human evaluators [36]. AI systems provide accurate and reliable interpretations of interproximal bone levels and radiographic bone loss, helping professionals identify areas of bone loss that could go unnoticed due to inexperience, fatigue, or lack of attention, thereby improving diagnostic accuracy [32]. These methods can offer dentists faster diagnoses, with radiograph evaluation times as brief as 1.2 s [23], and can also assess alveolar bone status following various periodontal therapies, both surgical and non-surgical [18]. Additionally, these algorithms can serve as a learning resource for inexperienced dentists and dental students [18,23].

The detection of periodontal bone loss through AI offers significant advantages, such as the ability to address the challenges faced by dentists and radiologists when interpreting images. Algorithms analyzing radiographs can distinguish anatomical structures with high precision, overcoming the limitations of the human eye in interpreting superimpositions, anatomical variations, and changes in the orientation of structures [40]. In this regard, Shan et al. [47], highlighted the versatility of AI in distinguishing anatomical structures and its usefulness in dentistry.

To distinguish different structures and lesions, the AI models used in the analyzed studies function, similarly, relying on CNNs or their variants. These networks are capable of classifying, detecting, and segmenting images: in classification, they identify which structure shows lesions; in detection, they locate the specific affected region; and in segmentation, they precisely delineate the edges of the lesion [48].

In addition, these algorithms have the ability to generalize results, allowing them to provide optimal performance when analyzing new data once trained with a dataset [42]. This is especially useful when conditions vary, such as when different X-ray techniques or equipment are used, or when applied to different populations, where it is crucial that a trained model be generalizable to other datasets [29]. The meta-analysis by Barbiero et al. [49], analyzes 109 datasets to understand what characteristics affect an AI model’s ability to generalize. One of the most relevant findings is that dimensionality, which represents the number of variables analyzed, does not negatively impact the generalization ability of models as much as traditionally believed [49]. It is also important that the data is diverse and well-distributed [28]. Additionally, the concept of “convex hull” is proposed to distinguish between interpolation, which refers to making predictions within the range of training data, and extrapolation, which involves making predictions outside that range [49]. This distinction is critical for evaluating the generalization capacity of models to different populations, as it helps identify whether the model performs adequately only in previously known conditions or if it can accurately handle unseen data [49].

### 4.5. Limitations of AI Methods

AI, despite being an optimal and innovative tool, also has limitations, including the need for large, well-labeled datasets of high-quality images [50]. To address this, augmentation procedures can be applied [35], which involve altering the dataset through transformations such as cropping, rotation, translation, zooming, elastic deformation, or changes in contrast or resolution [51]. The dataset is typically divided into training, validation, and testing sets [5,23,29,31,35,37,41,42,43,44]. The training set must exhibit minimal error and high precision, as its data serve as the reference for the AI learning process; if these labels are not accurate, the model will not learn properly. Therefore, it is crucial that the images are annotated exclusively by radiologists or dentists with extensive experience [52].

The sample size used for training, testing, and validation must be adequate, as otherwise, it calls into question the robustness and validity of the results obtained [53]. Some algorithms generate predictions without their developers being able to clearly identify how these decisions were made [43]. This is because these algorithms acquire knowledge and extract features from large datasets, rather than following pre-programmed instructions that can be easily traced back to specific rules or features [23,43]. This is referred to as the “black box” of AI [23,43,54,55]. For example, some models demonstrated high internal accuracy but lacked external validation, raising concerns about hidden dataset biases. Similarly, single-center datasets may reflect population-specific features, which limit generalizability when applied elsewhere.

The results obtained by AI architectures can also be subject to bias, which occurs when the training data do not adequately represent the target population or, conversely, include specific pathologies that hinder generalization to other datasets that do not have those characteristics [29,33,43,45]. Training data often do not adequately represent all populations, as they may contain specific features that make generalization difficult [56]. This can lead to incorrect diagnoses and amplify existing disparities [45,56]. For example, authors, such as Norori et al. [57], propose adopting open science practices, involving scientists and experts in the development of algorithms, to ensure that the data used fairly represent all populations [57].

It has also been observed that AI models face challenges when detecting bone loss in the very early stages, as they tend to show lower specificity in identifying incipient periodontitis [43]. This can be attributed to the inherent limitations of periapical images, which offer a reduced visual field, and the variable quality of the images, factors that hinder the early identification of subtle bone changes [47]. Potential strategies to address this include data augmentation to simulate incipient lesions, image preprocessing to improve contrast, and super-resolution methods to enhance subtle features.

### 4.6. Impact of AI on Clinical Practice

The use of AI in clinical practice can assist in early diagnosis and intervention in the field of periodontics [35]. The models could detect anything from a simple widening of the periodontal ligament to clearly visible lesions [58], but sensitivity and specificity values are not very high for the early stages of periodontal bone loss, as reflected by authors such as Icoz et al. [33].

In situations where the professional interpreting a radiograph lacks specific training to adequately assess the oral cavity, AI models can serve as “diagnostic support”, performing an initial screening and reducing the risk of interpretation errors [23].

AI can assist in periodontal treatment planning by providing a more precise and objective assessment of the severity and extent of bone loss [32]. These algorithms can also be used to monitor disease progression and evaluate the effectiveness of treatments at each patient visit, enabling more accurate and personalized follow-up [59]. In this way, AI facilitates more personalized dental care, adapting the diagnosis and treatment to the specific characteristics of each patient [59,60].

### 4.7. Perspective of Future Work

Deep learning systems, although recent, have significant room for improvement. To optimize their performance, larger studies are needed to increase their accuracy [44], along with multicenter studies to validate the system and use data from various sources across different centers, devices, and with diverse samples [23,44]. To enhance the performance of deep learning systems, it is essential to refine the input data, improve image quality, and optimize preprocessing processes to reduce noise and artifacts [23,32].

Another area for improvement is expanding the training dataset to include diverse and subtle lesions, which would allow the model to generalize better and reduce both false positives and false negatives [23,32]. Diagnostic results using deep learning could be enhanced by incorporating additional data, such as CBCTs, which provide information on bone loss volume, root morphology, and furcation involvement [18,35,39,40]. Furthermore, including variables such as systemic diseases (e.g., diabetes mellitus) and risk factors like tobacco use, along with complementary tests such as percussion and thermal and electrical tests, could improve diagnostic accuracy [18,23,35].

In future studies, it would be advisable to implement standardized acquisition protocols and restrict data collection to specific devices, controlling parameters such as kilovoltage, milliamperage, exposure time, and pixel size, to minimize variability in training data and improve the accuracy of the models [5,18,23,29,34]. Standardizing diagnostic metrics (e.g., sensitivity, specificity, F1 score, and AUROC) and harmonizing imaging protocols in future research would allow more meaningful benchmarking of AI performance.

Training models with data from a single institution or source, which may use specific equipment or protocols, can make the algorithms overly dependent on the particular characteristics of that center or device, limiting their ability to generalize to cases from other regions or hospitals that use different devices or imaging conditions [23,29]. Future studies should include data from multiple centers and X-ray equipment to improve the generalization and adaptability of the models [5,18,23,28,29,31,32,33,38]. 

The goal is to generalize AI models for bone loss detection, achieving precise and consistent results, regardless of the radiographs being analyzed [37].

Beyond 2D imaging, future research should explore AI integration with three-dimensional modalities such as CBCT, including low-dose protocols, as well as super-resolution imaging, to enhance volumetric assessment, furcation analysis, and early lesion detection. At the same time, challenges such as image artifacts, higher radiation doses, variability in acquisition parameters, and the lack of standardized annotated 3D datasets must be addressed. Preliminary studies have already explored AI applied to CBCT for periodontal applications, suggesting promising results for automated bone defect detection and segmentation, although these remain limited by small sample sizes and the lack of external validation [50].

Ethical and legal aspects must also be considered, including data privacy, transparency of algorithms, and medical liability, which are essential for the safe translation of AI into clinical practice. In real-world terms, this includes ensuring the secure handling of patient radiographs, auditing the quality of annotations provided by human experts during model training, and mitigating bias through external validation and explainable AI frameworks. A further challenge is clarifying responsibility in the event of an AI-related misdiagnosis, where liability may fall on the clinician using the system, the institution deploying it, or the developer of the algorithm, depending on existing regulatory frameworks [50,61]. Addressing these aspects will be critical for building trust and ensuring that AI can be responsibly integrated into periodontal diagnostics.

### 4.8. Limitations of the Present Work

One of the main limitations of this work was the heterogeneity. The heterogeneity across studies was multidimensional, including differences in imaging modalities (periapical, bitewing, and panoramic radiographs), CNN architectures (e.g., U-Net, YOLO, ResNet, ConvNeXt), dataset sizes (ranging from fewer than 100 images to more than 100,000), and the indices used to report performance (accuracy, sensitivity, specificity, F1-score, AUROC, DICE). This variability not only limits direct comparability between studies but also affects the generalizability of the findings. Additionally, the review was limited to 2D radiographic modalities; AI applications in 3D imaging (such as CBCT) were not included. Furthermore, the variability in imaging acquisition protocols and X-ray equipment affects reproducibility. A formal heterogeneity or sensitivity analysis was not feasible due to differences in imaging modalities, AI architectures, and outcome indices (e.g., sensitivity, specificity, F1 score, and AUROC), which limited direct comparability between studies. The lack of standardization and small sample sizes limit the generalization of the models. Although clinical interest in the technology is growing, many studies are retrospective and lack external validation, which could compromise their conclusions.

## 5. Conclusions

The conclusions of this systematic review highlight that AI for automated periodontal bone loss detection in radiographs is a promising and fast technique, but further studies are required to optimize its accuracy and clinical applicability. Advanced AI models have demonstrated superior diagnostic accuracy in detecting periodontal bone loss. These automated detection models can also identify other lesions, such as apical periodontitis, and accurately stage periodontitis, thus enhancing the overall diagnostic process. Additionally, AI helps reduce diagnostic errors related to fatigue or inexperience, enabling more precise and faster evaluations of periodontal bone status both before and after therapy.

## Figures and Tables

**Table 1:** Database search syntax.

Pubmed	((“Periodontal Diseases”[Mesh]) Or (“Alveolar Bone Loss”[Mesh]) Or (“Periapical Periodontitis”[Mesh]) Or (“Periodontal Bone Loss”) Or (“Alveolar Bone Resorption”)) And ((“Periapical Radiographs”) Or (“Dental Radiographs”) Or (“Dental Imaging”) Or (“Panoramic Radiography”) Or (“Orthopantomography”)) And ((“Deep Learning”) Or (“Convolutional Neural Networks”) Or (“Vision Transformer Networks”) Or (“Artificial Intelligence”) Or (“Automated Detection”) Or (“Machine Learning”))
Web of Science	((“Periodontal Diseases”) OR (“Alveolar Bone Loss”) OR (“Periapical Periodontitis”) OR (“periodontal bone loss”) OR (“alveolar bone resorption”)) AND ((“periapical radiographs”) OR (“dental radiographs”) OR (“dental imaging”) OR (“panoramic radiography”) OR (“orthopantomography”)) AND ((“Deep Learning”) OR (“Convolutional Neural Networks”) OR (“Vision Transformer Networks”) OR (“artificial intelligence”) OR (“automated detection”) OR (“machine learning”))
Scopus	TITLE-ABS-KEY (“Periodontal Diseases” OR “Alveolar Bone Loss” OR “Periapical Periodontitis” OR “periodontal bone loss” OR “alveolar bone resorption”) AND TITLE-ABS-KEY (“periapical radiographs” OR “dental radiographs” OR “dental imaging” OR “panoramic radiography” OR “orthopantomography”) AND TITLE-ABS-KEY (“Deep Learning” OR “Convolutional Neural Networks” OR “Vision Transformer Networks” OR “artificial intelligence” OR “automated detection” OR “machine learning”)
Cochrane	(“Periodontal Diseases” OR “Alveolar Bone Loss” OR “Periapical Periodontitis” OR “periodontal bone loss” OR “alveolar bone resorption”) AND (“periapical radiographs” OR “dental radiographs” OR “dental imaging” OR “panoramic radiography” OR “orthopantomography”) AND (“Deep Learning” OR “Convolutional Neural Networks” OR “Vision Transformer Networks” OR “artificial intelligence” OR “automated detection” OR “machine learning”)
Embase	(‘periodontal diseases’/exp OR ‘periodontal diseases’ OR ‘alveolar bone loss’/exp OR ‘alveolar bone loss’ OR ‘periapical periodontitis’/exp OR ‘periapical periodontitis’ OR ‘periodontal bone loss’/exp OR ‘periodontal bone loss’ OR ‘alveolar bone resorption’/exp OR ‘alveolar bone resorption’) AND (‘periapical radiographs’ OR ‘dental radiographs’ OR ‘dental imaging’ OR ‘panoramic radiography’/exp OR ‘panoramic radiography’ OR ‘orthopantomography’/exp OR ‘orthopantomography’) AND (‘deep learning’/exp OR ‘deep learning’ OR ‘convolutional neural networks’/exp OR ‘convolutional neural networks’ OR ‘vision transformer networks’ OR ‘artificial intelligence’/exp OR ‘artificial intelligence’ OR ‘automated detection’ OR ‘machine learning’/exp OR ‘machine learning’)

**Table 2:** Standardized table of the articles included in this systematic review.

Author and Year	Type of Study	Object of Study	Type of X-Ray	Sample Studied (Number of X-Rays)	Training, Testing, and Validation Data	Number of Patients	AI Technique Used	Reference Standard For Comparison	Index(es) Used for Model Quantification	Index Score
Kim et al. (2024) [28]	Diagnostic accuracy study	Classification of Periodontal Disease	OPG	100	N/S	N/S	UNet-CVAE	Overseen by study investigators	Accuracy	0.827
									Precision	0.696
									Sensitivity	0.794
									Specificity	0.842
Xue et al. (2024) [18]	Diagnostic accuracy study	Periodontal bone loss	OPG	320	(288, 32, N/S)	320	RNC	Dental care	F1 Score	Stage 1 91.78%
										Stage 2 82.90%
										Stage 3 92.71%
									Precision	89.45%
Boztuna et al. (2024) [23]	Diagnostic accuracy study	Bone loss in apical periodontitis	OPG	400	(340, 20, 40)	N/S	U^2^-Net (RNC)	Oral and Maxillofacial Surgery Resident and Dental Radiologist	DICE	0.788
									Intersection upon union	0.715
									Precision	0.776
									Recovery	0.854
									F1 Score	0.81
Liu et al. (2024) [29]	Diagnostic accuracy study	Bone loss in apical periodontitis	Periapical	1305	(3132, 200, 261)	N/S	YoCNET (Yolov5 + ConvNeXt) RNC	Dental Radiologists (3)	Accuracy	90.93%
									Precision	98.88%
									Sensitivity	0.8530
									F1 Score	0.9159
Nagareddy et al. (2024) [30]	Diagnostic accuracy study	Bone loss in apical periodontitis	Periapical	30	N/S	N/S	Diagnocat	Dental Radiologists (2)	Accuracy, sensitivity, specificity, confidence interval, and correlation of the two radiologists with AI	Sensitivity R1 93.8%/R2 83.3%/AI 86.5% Specificity R1 96.7%/R2 80%/IA 88.1% R1 (84.8 ± 8.76)/R2 (84.2 ± 7.74)/AI (86.5 ± 9.18) confidence interval Correlation with AI: R1 0.383 and R2 0.347
Kong et al. (2023) [31]	Diagnostic accuracy study	Periodontal bone loss	OPG	1747	Random Split	N/S	Two-stage RNC-based Periodontitis Detection Network (PDRNC)	Dental care	F1 Score	No injuries 0.929
										Mild 0.051
										Severe 0.020
									Precision	Accuracy: 0.762
Chen et al. (2023) [32]	Diagnostic accuracy study	Periodontal bone loss	Periapical and bite fins	8000	N/S	270	RNC with VGG-16 and U-Net architecture	Experienced dentists specializing in periodontics and radiology	Precision	97.0%
Icoz et al. (2023) [33]	Diagnostic accuracy study	Bone loss in apical periodontitis	OPG	306	N/S	N/S	YOLO-based computer-aided diagnosis (CAD) (RNC)	Dentists, maxillofacials and radiologists	Accuracy (PPV)	Clearly visible apical periodontitis (0.93 sensitivity, 0.96 F)
									Recovery (sensitivity)	Clearly visible apical periodontitis in the mandible (0.93 sensitivity, 0.96 F)
Ayyildiz et al. (2023) [34]	Diagnostic accuracy study	Periodontal bone loss	OPG	2533	(2026, 506, N/S)	N/S	RNC-based autonomous transfer (TL) learning methods	Experienced dentists	Accuracy	0.907
									AUROC	0.888
									Specificity	0.944
									Precision	0.88
									Sensitivity	0.883
									F1 Score	0.856
Chen et al. (2024) [35]	Diagnostic accuracy study	Periodontal Bone Loss and Classification of Periodontal Disease	Periapical	336	(82, 336, 20)	N/S	Mask-RRNC and U-Net (RNC)	Three periodontists	Diagnostic accuracy	72.80%
									AUROC	0.946
									F1 Score	0.891
									Sensitivity	0.88
									Specificity	0.906
Saylan et al. (2023) [5]	Diagnostic accuracy study	Periodontal bone loss	OPG	685	(549, 68, 68)	N/S	YOLO-v5 (RNC)	Oral and maxillofacial radiologist and periodontist	Sensitivity	0.75
									Precision	0.76
									F1 Score	0.76
Li et al. (2022) [36]	Diagnostic accuracy study	Caries and periodontitis	Periapical	4129	N/S	4525	Modified ResNet backbone	Experienced dentists, junior, and a computer scientist	F1 Score	F1: 0.8283
									Sensitivity	SEN: 0.8200
									Specificity	SPEC: 0.8400
									VPP	PPV: 0.8367
									VPN	NPV: 0.8235
Tsoromokos et al. (2022) [44]	Diagnostic accuracy study	Periodontal bone loss	Periapical	446	(327, 70, 49)	54	CNN	Dental care	Sensitivity	0.96
									Specificity	0.41
									Accuracy	0.80
Alotaibi et al. (2022) [37]	Diagnostic accuracy study	Periodontal bone loss	Periapical	1724	(1206, 173, 345)	1610	RNC	Experienced dentists and a periodontist	Precision	Accuracy, recovery score F1 binary rating >70%
									F1 Score	F1 45–70%
										Bone Loss Prediction 0.75
										Mild bone loss 0.45
										Normal bone levels 0.70
									Sensitivity	0.73
									Specificity	0.79
Kearney et al. (2022) [38]	Diagnostic accuracy study	Periodontal bone loss	Periapical and bite fins	103,914	(80,326, N/S, 12,901)	10,489	Generative Partial Convolution Adversarial Restoration (RNC) Network	A periodontist and two general dentists	Absolute Mean Error	1.5 mm
Zadrożny et al. (2022) [39]	Diagnostic accuracy study	Missing teeth, caries, fillings, prosthetic restorations, root canals, residual roots, periapical lesions, and periodontal bone loss	OPG	30	N/S	30	Diagnocat (RNC)	Three dentists	Sensitivity, specificity	Periapical lesions: AI sensitivity (0.390), IA specificity (0.981)
										Periodontal bone loss: Sensitivity IA 0.801; AI specificity 0.847;
Danks et al. (2021) [40]	Diagnostic accuracy study	Periodontal bone loss	Periapical	340	N/S	63	Deep neural network with hourglass architecture (RNC)	Two periodontists in postgraduate studies	Percentage of correct Keypoints (PCK), error level, and accuracy level	PCK: 88.9% (one root), 73.9% (two roots), 74.4% (three roots), 83.3% (all three root types together)
										Periodontists’ assessment error: 10.69% ± 9.15
										Periodontist accuracy level: 58%
Bayrakdar et al. (2020) [41]	Diagnostic accuracy study	Periodontal bone loss	OPG	2276	(1856, 210, 210)	N/S	RNC Google Net Inception v3	Oral and maxillofacial radiologist and periodontist	Sensitivity	0.9429
									Specificity	0.8857
									Accuracy	0.8919
									Precision	0.9143
									F1 Score	0.9167
Verma et al. (2020) [42]	Diagnostic accuracy study	Cavities, periapical lesions, alteration of alveolar bone height, and impactions of the third molar	OPG	366 (increased to 1098)	(878, 220, 87)	N/S	RNC and SVM (Support Vector Machine)	Dentist, Dental Radiologist	Precision	0.9869
									Specificity	0.9857
									Sensitivity	0.9795
Kim et al. (2019) [43]	Diagnostic accuracy study	Periodontal bone loss	OPG	12,179	(11,189, 800, 190)	N/S	DeNTNet	Dental care	F1 Score	0.71
									Sensitivity	0.87
									AUROC	0.95
									Specificity	0.96
									VPP	0.6
									VPN	0.97

N/S—Not specified. OPG: Orthopantomography. Sensitivity: True positive rate. Specificity: True negative rate. Precision/PPV/VPP: Positive predictive value. NPV/VPN: Negative predictive value. F1 Score: Harmonic mean of precision and sensitivity. AUROC: Area under the ROC curve. IoU: Overlap between predicted and true regions. DICE: Similarity measure between segmentations. PCK: Percentage of correct key points. Recovery: Synonym for sensitivity. Absolute Mean Error: Average deviation from the true value.

**Table 3:** Cohen’s Kappa index (κ) of the interrater concordance analysis for the QUADAS-2 test.

	Risk of Bias				Applicability Concerns	
QUADAS-2 Category	Patient Selection	Index calculated	Reference Standard	Flow and timing	Patient Selection	Reference Standard
Cohen’s Kappa (κ)	0.8864	0.8276	0.7727	0.7727	0.7727	0.6429
